# Metagenomic analysis of heavy water-adapted bacterial communities

**DOI:** 10.1099/mgen.0.001414

**Published:** 2025-05-29

**Authors:** Juan Rivas-Santisteban, José M. Martínez, Cristina Escudero, Rebecca Hernández-Antolín, Laura Cid-Barrio, Gary J. Ellis, Esteban Domingo, Carlos Sánchez, Francisco Sobrino, Ricardo Amils

**Affiliations:** 1 Department of Systems Biology, Centro Nacional de Biotecnología (CNB-CSIC), Madrid, Spain; 2 Centro de Biología Molecular Severo Ochoa (CBMSO-CSIC), Universidad Autónoma de Madrid, Madrid, Spain; 3 Department of Geoscience, Eberhard-Karls-University, Tuebingen, Germany; 4 Laboratorio Subterráneo de Canfranc (LSC), Huesca, Spain; 5 Instituto de Ciencia y Tecnología de Polímeros (ICTP-CSIC), Madrid, Spain; 6 MIRE Group, Department of Materials Physics, Faculty of Sciences (UAM), Madrid, Spain

**Keywords:** deuterium oxide, deuterophiles, deuterophilic bacteria, heavy water, heavy water adaptation, limits of adaptation, metagenomics

## Abstract

Micro-organisms can survive and thrive in unusual and extreme environments. Here, we present a metagenomic analysis of living bacteria found in highly pure, deleterious heavy water (>99% D_2_O), stored in sealed plastic containers for more than 30 years, without any external supply. Deep DNA sequencing analyses have revealed that the most abundant genetic signatures were primarily associated with *Pseudomonadota* and *Bacteroidota*. These bacteria exhibited shorter gene lengths and depletion of polar and metabolically costly amino acids compared to the related species from light water environments. Genes related to DNA transposition, repair and modification were notably abundant, particularly mutant forms of the IS3 transposable elements family. We also explore potential carbon and energy sources and discuss the evolutionary implications of bacteria capable of surviving in such an extreme human-made environment.

**Impact Statement**

We present the first metagenomic analysis of viable bacteria growing in pure heavy water. High concentrations of deuterium are known to exert deleterious effects on living systems. Although sensitivity varies among taxa, it has long been thought that the adaptation and maintenance of complex metabolisms to fully deuterated water was precluded by isotopic effects, e.g. greater stability of hydrogen bonds. This intuition has been supported by many deuterium enrichment experiments where only phenotypic responses were observed. However, this view is largely challenged by our finding of viable bacteria in three separate high-density polyethylene containers, filled with pure D_2_O, and kept sealed for more than three decades. Thus, we here provide strong evidence for genotypic adaptation and the presence of complex metabolic networks within bacterial communities grown and maintained in pure heavy water. The fact that this system is viable even under oligotrophic conditions suggests that at least some bacteria can easily adapt to thrive in pure heavy water.

## Data Summary

Raw reads will be available at the Sequence Read Archive (SRA) BioProject PRJNA1125856 upon publication. The summary metagenomic table and the raw reads are available at https://doi.org/10.5281/zenodo.14610112. Supplementary information and figures are attached, while supplementary files and tables can be found at https://doi.org/10.5281/zenodo.15118621.

## Introduction

Biological reactions predominantly occur and evolve in protium (^1^H, hereafter denominated H) water. While natural waters contain varying proportions of deuterium (^2^H, hereafter denominated D) [1], these fluctuations – driven by seasonal abiotic factors [2] – typically remain close to the value of $3\times 10^{-4}$ by weight, where DHO is the most prevalent deuterated molecule [3]. The Earth’s average D/H ratio is very low (≈1 : 5,700) and is unevenly distributed across different water bodies – with, for example, Arctic waters showing approximately half the global average [4]. Due to this scarcity of D in natural waters, our understanding of the physiological adaptations to deuterium-enriched environments has historically been limited.

However, during the past century, several key studies on artificial media containing deuterium oxide (D_2_O, also termed heavy water) have emerged. These studies consistently demonstrated that living beings can variably adapt to and thrive in the presence of deuterium oxide [5]. Mosin *et al.* first proposed that as organisms increase in metabolic complexity, their ability to tolerate the deleterious effects of higher deuterium concentrations decreases [4,6]. Micro-organisms, for example, display significant variability in their tolerance to deuterium, ranging from 95% [D] (w/v) for *Haloarchaea* to only 70% [D] (w/v) for the algae *Chlorella vulgaris* [4,7]. However, despite high levels of deuterium incorporation, biosynthetic rates consistently decrease upon exposure to D_2_O [8,10]. This reduction may stem from the impaired function of some proteins due to isotope effects, as deuteration can alter binding affinities and slow the rate of some spontaneous biochemical reactions, while increasing the velocity of others [11]. Wiberg described the isotopic effects of deuterium, noting that its bonds should be more stable due to a lower zero-point vibrational energy, meaning a reduced chemical reactivity [12]. Indeed, experiments have shown that deuterated proteins have increased stability in the *α*-helix domains [13]. It follows that any cellular physiology evolved in natural environments must respond to the D_2_O-evoked stress by adapting its genes, their expression, or both [14].

Previous studies have identified the presence of bacteria from various genera inhabiting heavy water taken from spent nuclear fuel pools [15,16]. Instead, here, we show the results of the metagenomic survey of bacterial communities retrieved from three highly pure D_2_O independent sources, revealing notable differences from their counterparts inhabiting standard water environments.

## Methods

### Detection of viable bacteria in heavy water (D_2_O)

The three high-density polyethylene (HDPE) containers analysed in this work were donated by CERN to C. Sánchez in 1989 as part of support to the experiments conducted at the Universidad Autónoma de Madrid. Since then, the containers have been kept sealed, protected from any environmental influence. These drums contained slightly different, yet extremely high, D_2_O purity levels (container 1: 99.77%, $\rho =1.097\;\text{g}\times \;{\text{cm}}^{-3}$; container 2: 99.4%, $\rho =1.093\;\text{g}\times \;{\text{cm}}^{-3}$; container 3:≈ 100%, $\rho =1.102\;\text{g}\times \;{\text{cm}}^{-3}$). The deuterium purity has also been determined by inductively coupled plasma MS, being the main impurities Si, Li, Na, K, B and Ca. Concentrations of those elements were in the range of a few hundreds of microgrammes per litre (Fig. S1, available in the online Supplementary Material). The presence of bacteria in highly pure D_2_O was first discovered in recent years by serendipity, leading to subsequent analyses. Samples were transferred from the plastic containers (>10 l per container) to glass ISO flasks (Pyrex, 3×1 l volume), in which they were kept for an additional 11 months in darkness. Microbial cell counts were carried out by fluorescence microscopy. Cells were hybridized *in-situ* (FISH) with CY3-labelled EUB 338 (I-III) and ARC915 probes and counterstained with SYTO9 and SYBR Gold following the previously described protocols [17]. The samples were imaged using a confocal laser scanning microscope LSM710 coupled to an AxioObserver microscope (Carl Zeiss, Germany) and equipped with diode (405 nm), argon (458/488/514 nm) and helium and neon (543 and 633 nm, respectively) lasers. Images were captured with a 63×/1.4 oil immersion lens.

### DNA extraction and sequencing

Environmental DNA was extracted from 100 ml of D_2_O subsamples collected from each drum using the DNeasy PowerSoil Pro Kits (Qiagen), following the manufacturer’s instructions. A negative control was included during DNA extraction. DNA concentration was determined fluorimetrically using the Qubit 1X dsDNA HS Assay kit (Thermo Fisher). DNA samples were subjected to next-generation sequencing (MicrobesNG, UK). Genomic DNA libraries were prepared using the Nextera XT Library Prep Kit (Illumina, San Diego, USA) according to the manufacturer’s protocol, with the following modifications: input DNA was increased twofold, and PCR elongation time was extended to 45 s. DNA quantification and library preparation were performed on a Hamilton Microlab STAR automated liquid handling system (Hamilton Bonaduz AG, Switzerland). The libraries were sequenced on an Illumina NovaSeq 6000 (Illumina) using a 250 bp paired-end protocol.

### Metagenomic analysis

The raw sequencing of each sample was processed as a co-assembly using the default features of the SqueezeMeta pipeline [18]. The following software was used with standard parameters, unless specified otherwise. We limited the contig size to 200 bp using Megahit [19]. Aragorn software was used for t/tmRNA prediction [20], and ORF prediction was carried out using Prodigal [21]. Homology search against NCBI taxonomic and functional databases was performed with DIAMOND v.0.9.13 [22], without specifying a minimum or maximum *e*-value for similarity. For further functional annotation, HMMER [23] was used to search against the Pfam, COG and KEGG [24,26] databases. Binning procedures were conducted using both CONCOCT and metaBAT [27,28], obtaining the best sequences from both datasets with DASTool software [29], all with the latest versions matching the SqueezeMeta release. Abundance estimation was automatically calculated with SqueezeMeta v.1.5.1. Gene abundances were expressed as transcripts per million (TPM): the sum of all gene abundances in a sample equals 1 million TPM. ORF retrieval and some of the abundance information displayed in plots were achieved using the SQMtools v.1.6.3 package for R [30]. Metagenomic processing for the Malaspina dataset [31] (accessible at https://www.ebi.ac.uk/ena/browser/view/PRJEB27154), used for comparison, was performed using the same pipeline, but instead of co-assembly, a sequential analysis was used due to the large number of used samples (75).

### Predicted average gene length

To estimate the average gene length in the three containers, we have calculated the mean (weighted by the relative abundance) and the sd in bp of the predicted coding sequences (CDSs) for each of the containers, as follows:


$$\frac{{\text{CDS}}_{i}\left(\text{bp}\right)\times {\text{TPM}}_{i}}{\bar{x}\;\text{TPM}}$$

which is consistent with the co-assembled nature of the metagenome. As a precaution, only CDSs with start and stop codons were considered. In addition, we used both CDSs with and without gene annotations to avoid introducing bias towards the gene size based on the genes that are known.

### Functional delineation of proteins via placement

Reference phylogenetic trees were constructed using the IQ-TREE software [32] for the above retrieved sequences, as per Rivas-Santisteban *et al.* [33]. These trees act as classifiers of metagenomic sequences, helping to discern truly functional sequences from similar paralogues. The tested genes were retrieved from the metagenomes using the pertinent hidden Markov model [34] from Pfam, COGs and/or KEGG databases for each gene. Then, we identified truly functional sequences by propagating the functional annotations (displayed in Fig. S2) onto the reference trees. These candidate sequences were placed in the pertaining reference tree to distinguish particular sequences with biochemical evidence from the literature. We tagged the trees and placements using MetaTag v.0.1.1 [35].

### Estimation of $k_{a}/k_{s}$ ratios

To assess the possible fit between known orthologues and the genes retrieved from heavy water samples, we applied the estimates of Nei and Gojobori [36] and those provided by PAML v.4 software [37], which is based on the maximum likelihood algorithm.

### Assessment of protein diversification

We assessed the expected sequence richness by fitting


$$d_{\text{exp},s}=c_{s}A_{s}^{\gamma_{s}}$$

where s represents the sequences (ORFs) considered for a taxon, implementation and environment, abundance (A) was inferred using TPM, where $c_{s}$ and $\gamma_{s}$ were constants inferred from the natural number of ORF variants (dobs). Thus, δ was calculated as a coefficient between dobs and dexp. The kernel density estimates shown were obtained with custom Python software. More details about this metric can be found in [33].

## Results and discussion

### Viability and metagenomic composition of bacteria after prolonged survival in heavy water

**Fig. 1.**
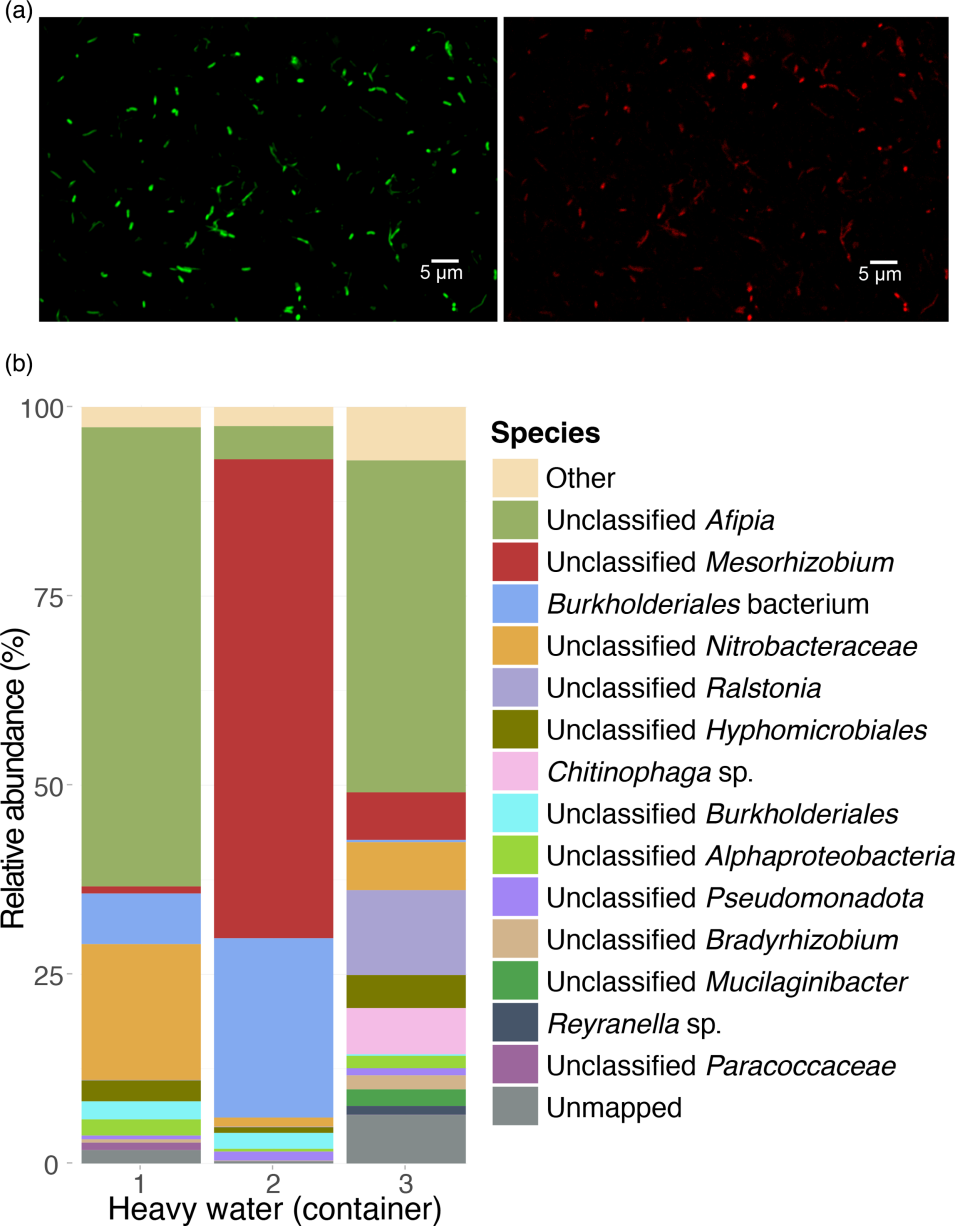
Taxonomic composition of the bacterial communities found within three flasks of highly pure heavy water. (**a)** Living bacteria recovered from the samples by filtration and detected by FISH. Green, DNA general staining with SYBR Gold; red, EUB 338 FISH probe signal (scale bars, 5 µm; Methods). (**b)** Taxonomic assignments from metagenomic output in relative units.

The opportunity to characterize microbial populations that had remained undisturbed in highly pure D_2_O environments arose when living bacteria were discovered within three HDPE containers that had been sealed for 30 years. The purity of the D_2_O in these containers was estimated to be 99.76±0.3% (range 99.4–100%; see Methods). Micro-organisms present in the containers ranged from $6\times 10^{5}$ to $7\times 10^{6}$ bacterial counts per millilitre. Since the signal intensity of FISH is directly proportional to the number of ribosomes present per cell (minimum of 370 ribosomes/cell [38] required for detection), we applied this technique to assess the viability of the micro-organisms inhabiting the containers. Positive results from the general bacterial probe (EUB 338 I-III) indicated that at least 35–55% of the cells were not only alive but also metabolically active (Fig. 1a). To further analyse these microbial populations, we concentrated bacteria from samples of each of the three HDPE containers and extracted DNA. The total DNA concentration, which was consistent with the levels found in other oligotrophic environments, was determined to be 30 ng ml^−1^ prior to the amplification and sequencing protocol. We sequenced and co-assembled DNA using Illumina technology and the bioinformatics pipeline described in Methods. A total of 162 species tags were identified across 6,527 co-assembled contigs. Of the 12 bins retrieved, four represented high-quality metagenome-assembled genomes (MAGs; >90% completion and <10% contamination). The number of contigs in each bin was highly variable (40–2,106), with the maximum size of high-quality MAGs never exceeding 145 contigs (Table S1).

Bacteria largely dominated the samples. The presence of at least three dominant phyla (*Pseudomonadota*, *Actinobacteriota* and *Bacteroidota*) suggests a broad adaptability of bacteria to the heavy water environment. *Afipia* and *Mesorhizobium* (*Pseudomonadota*) were the most prevalent genera identified in the three containers. Other taxa were present in one or two of the three containers (Fig. 1b and Table S2). Species *α*-diversity was similar in containers 1 and 2 and showed a known-species richness of 61 in each (170 and 168 including unknowns, respectively). The *α*-diversity of container 3 was higher, with a 126 known-species richness (278 including unknowns). *β*-Diversity, based on the presence or absence of species among communities, may be approximated, when there is no spatial overlap data between species, using Jaccard’s index [39]. It was estimated at 0.458 between containers 1 and 2, 0.302 between containers 1 and 3 and 0.285 between containers 2 and 3. These results show that highly pure heavy water does not severely restrict the development of different bacterial communities, since these values of *β*-diversity were moderate to high for the same kind of environment [40].

### Growth of heavy water-isolated bacterial communities in deuterium-enriched media

**Fig. 2.**
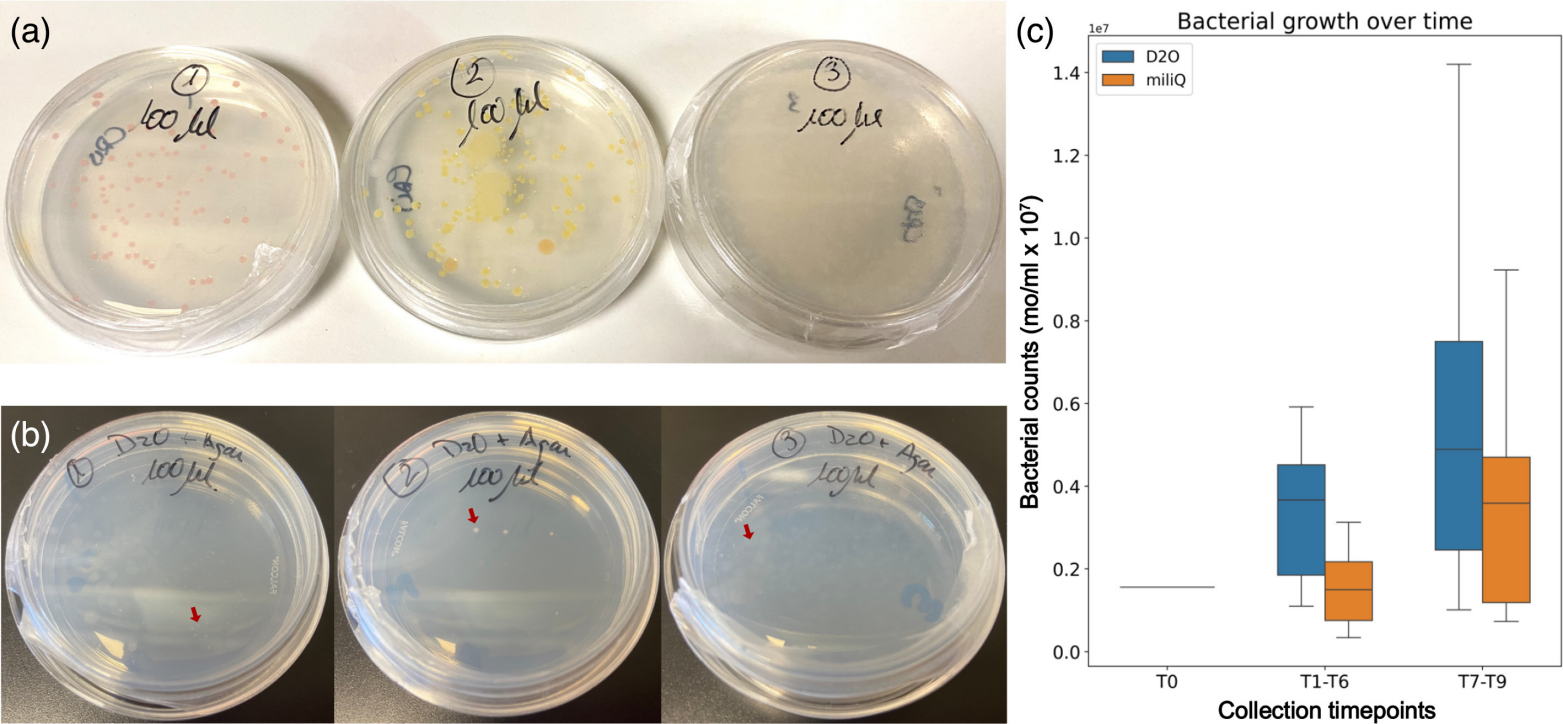
Bacterial growth in heavy water media. (**a)** Isolated bacterial colonies in oligotrophic media+100% D_2_O. Colonies with variable colours were isolated from the different containers. (**b)** Isolation attempts from each oligotrophic culture (agar+100% D_2_O). The red arrows show an example of isolated colonies. (**c)** Comparative growth over time (estimated by micro-organism count per ml, $mo/ml\times 10^{7}$). T1–T6 : 220 days, *n*=6. T7–T9 : 198 days, *n*=3. While the growth in heavy water remains nonsignificantly different to the growth in Milli-Q water, the average growth in heavy water was consistently higher, evidencing a notable effect size.

To evaluate the growth potential of the inferred bacterial communities, we cultured bacteria from each container using Reasoner’s 2A agar, an oligotrophic medium designed for slow-growing, common water-associated bacteria [41], combined with highly pure heavy water (≈100% D_2_O). Differently coloured colonies and growth patterns were recovered from containers (Fig. 2a). These colonies were further subcultured exclusively in highly pure heavy water with sterile agar. Although some small colonies were successfully obtained (Fig. 2b), nutrient limitations became evident, as repeated transfers eventually led to no further growth. Nevertheless, we were able to extract and sequence 16S rRNA genes from these transient isolates (File S1), identifying genera also detected in the metagenomic dataset – mainly *Ralstonia* spp. and *Sphingomonas* spp.

In parallel, we performed a comparative growth assay where a diluted inoculum from the original containers (1 : 10) was grown in Milli-Q water or pure heavy water (Fig. 2c). A trend in cell counts compatible with slow bacterial growth was observed. Over an incubation period of 418 days, no statistically significant differences in growth were detected between treatments (p=0.246). However, a notable difference in average bacterial counts ($-2.37\times 10^{6}$) is reported, suggesting a potential trend towards enhanced growth in heavy water.

### General genomic features of heavy water-isolated bacteria

The total metagenomic G+C content averaged 66.3%, placing it at the higher end of the typical range in prokaryotes (16–77% [42]). This G+C value correlates with low metabolic cost aa usage [42]. In contrast, the weighted average gene length was 781±61 bp (798±83 bp for the most abundant MAGs, corresponding to *Afipia* spp.; Methods), which represent low values. For reference, the average gene length for prokaryotes is 924±9 bp [43] and 875 bp for *Afipia septicemium* [44]. The maximum gene length observed in our dataset was 8,748 bp (*bmaC* gene), while the minimum was 60 bp, a size shared by many genes. Thus, this low average is not due to a narrow range but rather a skewed distribution. Finally, the average genome size was 4.68±1.06 Mb, which is consistent with expectations for bacteria [45]. However, the genome size of the most abundant MAGs tended to be shorter compared to reference values for their closest relatives (Table 1). Although no oligotroph could be confirmed, and the G+C content is typically lower for oligotrophs [46,47], these results may reflect the adaptation of bacteria through genome streamlining towards energy efficiency.

**Table 1.** Reference genome sizes of various species in comparison to the best quality MAGs and bins retrieved from pure heavy water (ordered by abundance)

MAG	Genome size (Mb)	Completeness (%)	Reference genome size (Mb)
*Mesorhizobium* sp.	6.02	97.38	*Mesorhizobium japonicum*=7.02 [65]; avr.≈8 [66]
*Afipia* sp. 1	4.95	97.81	*Afipia birgae*=5.32 [67]
*Afipia* sp. 2	5.08	93.16	*A. septicemium*=5.08 [44]
*Burkholderiales* sp.	5.51	99.31	*Burkholderiales* avr.=5.85±10.72 [68]
*Ralstonia* sp.	3.64	71.65	*Ralstonia* avr.=5.22±0.3 [69]
*Chitinophaga* sp.	2.96	76.29	*Chitinophaga* avr.=7.51 [70]

Completeness refers to how much of a particular genome has been reconstructed from the metagenomic data. If a genome is 100% complete, it means that all expected genes and genomic regions for a taxon are present in the assembly. *Burkholderiales* spp., *n*=190 (supplementary of [68]); *Ralstonia* spp., *n*=18 (supplementary of [69]); *Chitinophaga* spp., *n*=47 (main of [70]).

The proportion of predicted ORFs without known homologues (orphans) was found modest (5.95%) [48]. A summary of these predictions is provided in Table S3, while the most common KEGG and Pfam functional predictions are shown in Fig. S3. The most abundant functions were also ubiquitous, although there were slight functional differences between containers, both in the quantity and type of predicted ORFs. For example, peptide/nickel permease orthologues were consistently more abundant in the second drum, as carbon monoxide dehydrogenases were in the first. This variation may reflect the taxonomic diversity mentioned earlier. Overall, these results broadly suggest that highly pure heavy water does not extremely limit the bacterial functions, or the species that may thrive there, contrary to previous assumptions.

### Prevalence of DNA modification and repair genes in heavy water bacteria

Transposases and branched aa transporter subunits were the most frequently predicted proteins across containers (Fig. S3). Indeed, transposases are the most common genes in the biosphere, with an estimated average gene count percentage of 0.83% per genome (*n*=693 [49]). The average metagenomic abundance of transposases is 4,026.17 TPM (*n*=178) [49]. However, in D_2_O-retrieved metagenomes, we found this value to be doubled or tripled (2.71%±0.75% of the counts based on a conservative estimate; Methods), and the normalized counts averaged 11,390.64 TPM ±4,294.21.

Closely related to DNA modification by transposition are the mechanisms of informational repair. In particular, some specific DNA repair genes were abundant, including *alkA*, *ada* and *luxA*, which are related to oxidative DNA damage [50], and they have been reported to increase their expression in *Escherichia coli* when exposed to deuterium [51]. Heavy water diminishes the expression level of the *katG* gene in *E. coli*, potentially resulting in hydrogen peroxide (H_2_O_2_) accumulation, and consequently, DNA damage, as indicated by increased expression of the *recA* (recombinase) gene [52]. In this regard, we confirmed the consistent presence of these genes in D_2_O-retrieved metagenomes: *ada*/*alkA* (with 3-methyladenine DNA glycosylase activity) and *katG* (Table 2). We hypothesize that this represents a molecular mechanism for deuterium-induced differential mutagenesis (as observed by De Giovanni [14]) via downregulation/upregulation of *katG*/*ada-alkA* and *recA* genes. We provide the basis for this hypothesis in the supplementary information. A more detailed summary of the DNA repair functions found is provided in Fig. S4.

**Table 2.** Link between the DNA repair and SOS signalling genes found and their expression when exposed to deuteration

Gene	Molecular function	Drum 1	Drum 2	Drum 3	Expression in D_2_O
*ada*/*alkA*	nt excision [71]	334.44	406.52	318.108	↑↑↑ [51]
*recA*	Double-strand repair [72]	255.76	219.37	221.01	↑↑ [52]
*luxA*	*alkA* signal processing [51,73]	0	0	13.667	↑ [51]
*luxQ*/*luxR*/*luxO*	Signal regulators [74]	12.14	103.37	5.38	No data
*katG*	Catalase-peroxidase [75]	165.91	147.59	203.16	↓↓↓ [52]
*katE*	Catalase (signalling) [76]	176.55	16.09	64.45	No data

In each drum, the value of abundance is indicated as TPM (here synonym to FPM). The last column qualitatively represents the reported increase (arrow up) or decrease (arrow down) expression in gene expression when exposed to deuterium oxide (e.g. *katG* is extremely repressed).

### Evidence of adaptation in transposases recovered from heavy water bacterial communities

**Fig. 3.**
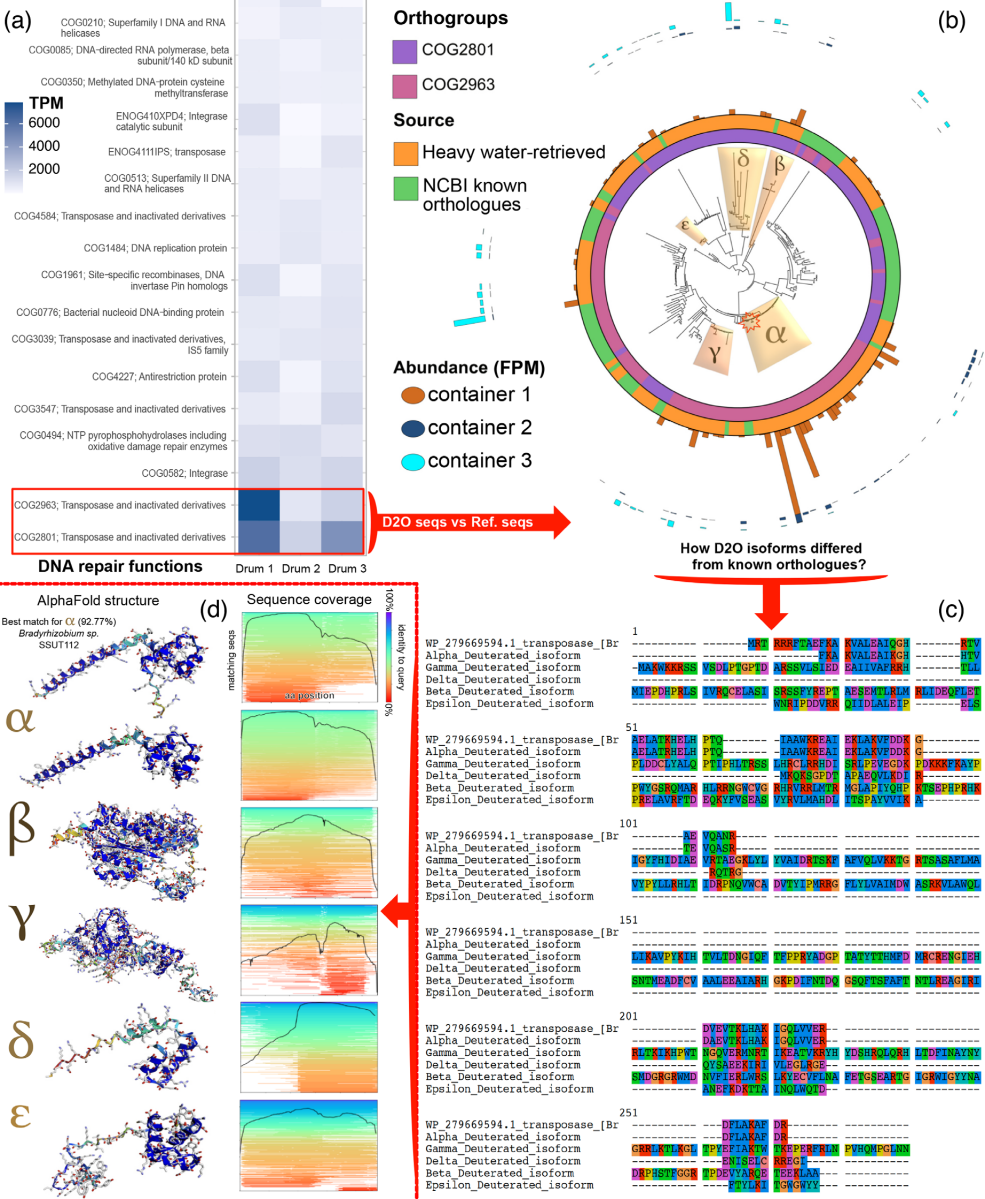
Divergence in heavy water-retrieved isoforms of highly abundant functions (IS3-like transposases) may reflect characteristic genotypic adaptation. (**a)** Top orthogroups related to DNA repair. COG2801 and COG2963 stand out as putative IS3 transposases. (**b)** Phylogenetic reconstruction for reference orthologues and problem sequences. Five different dissimilarity groups between D_2_O-retrieved (orange) and reference (green) sequences are observed. We ordered them by their abundance. The deuterium-retrieved transposase group *α* was the most abundant across containers (red star). (**c)** Alignment of a known transposase against D_2_O-retrieved representatives. When considering all abundant D_2_O-retrieved transposases (*n*=129) vs those from common sources (*n*=70), these aa substitutions were biassed towards enrichment of aromatic residues (Fig. S6). Then, we visualized the structures predicted by AlphaFold. (**d) **On the left, there are notable differences in the structure of the short domains. Whether these are due to adaptation to heavy water or deuteration is yet to be interrogated. Their coverage (shown at the right of each structure) was found to be variable.

An intriguing question to explore is whether the overrepresentation of transposase genes reflects an adaptive process (Fig. 3a). Transposition induces mutations and rearrangements that are unlikely to be caused by other mechanisms [53]. The large number of *putative transposase* annotations found mainly belongs to the IS3 family (copy-out–paste-in transposons). A phylogenetic reconstruction using these sequences (orange in Fig. 3b) and the corresponding reference sequences (green in Fig. 3b), both belonging to the COG2801 and COG2963 orthogroups (purple and pink in Fig. 3b), revealed that branches from different sources rarely mix. This may highlight the emergence of new clades adapted to heavy water. To facilitate comparison between these clades and the known orthologues, we selected a representative sequence from each clade – the longest and most abundant sequences. For the known orthologues, we chose a reference sequence from *Bradyrhizobium* sp., which had the best blastp match (92.77% identity). Multiple sequence alignment revealed preliminary structural and compositional differences between the heavy water-retrieved sequences and the known reference (Fig. 3c). Similarly, the AlphaFold structures [54] were dissimilar, suggesting exaptation to the isotopic effects outlined (Fig. 3d). To investigate differences in aa usage, we performed correlation analyses and unpaired t-tests on the relative aa composition of the D_2_O-retrieved transposases (*n*=129) and those from common sources (*n*=70, Table S4 and File S2). While the aa compositions of common transposases self-correlated slightly, D_2_O-retrieved transposases showed a stronger self-correlation (Fig. S5). We found that five aa were significantly enriched, nine were significantly depleted and six had the same incidence (Fig. S6). These differences involved a higher degree of aromatization and a lower metabolic cost [55].

**Fig. 4.**
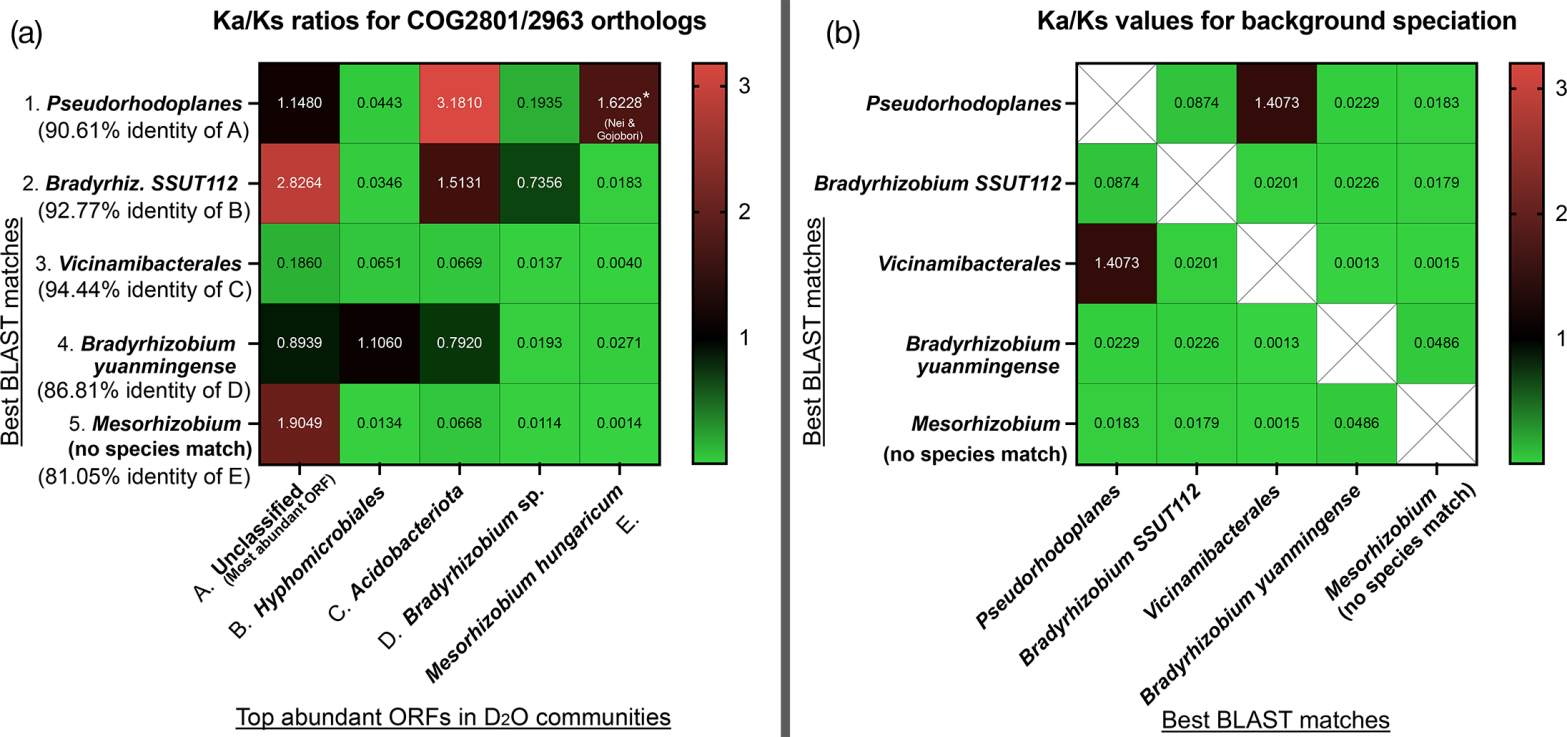
$k_{a}/k_{s}$ ratios between the most abundant transposases found in the three heavy water samples and their closest orthologues in public databases. Since this ratio accounts for the type of substitution that the sequences have undergone for each synonymous or non-synonymous site, it is widely used to approximate whether the change between orthologous proteins recalls the conservation of function under purifying selection (when $k_{a}/k_{s}\approx 0\leq 1$ the mutational space is constrained), whether the changes are likely neutral ($k_{a}/k_{s}\approx 1$) or whether changes are positively selected (when $k_{a}/k_{s}§amp;gt;1$ proteins diverged quickly, yet adaptively). (**a)** D_2_O-retrieved transposases vs best taxon-wise blastp matches. A to E are ordered by their relative abundance in the containers. A is the match for 1, B for 2 and so on. Positive selection processes occurred frequently and very noticeably between reference transposases and D_2_O-retrieved transposases, suggesting rapid functional exaptation. Green, purifying selection; black, neutral divergence; red, positive selection. (**b)** The instances of positive selection described cannot but marginally be explained by speciation processes, since the reference vs reference comparison showed a strong purifying selection in almost all cases (IS3 transposases are preserved).

To test whether the reported changes in sequence and aa composition in transposases were adaptive, we made the basic assumption that the founder bacterial communities had a prior evolutionary history in standard water media. $k_{a}/k_{s}$ ratios [56] for the transposase genes were calculated relative to the most identical transposase sequence recorded in data banks. This procedure was restricted to the most similar protein match of the closest taxon (GenBank accession numbers and sequences available in File S1). The result (Fig. 4) was significant for adaptive change in the most abundant D_2_O-retrieved transposases (red and black boxes of Fig. 4a). In contrast, divergence due to speciation (time-dependent events [57]) could not explain the observed adaptive changes, as the transposases retrieved from different taxa in standard media were mostly under strong purifying selection (green boxes in Fig. 4b). Ratios for other genes, such as *ada*/*alkA* and *accB* (acetyl-CoA carboxylase subunit), showed weaker indications of adaptive change (Fig. S7), a result expected for metabolic genes that are deleterious upon subfunctionalization [58].

### Metabolic features of D_2_O-retrieved metagenomes

#### Carbon metabolism

Most parts of the known carbon fixation pathways [59] were at least partially represented in our metagenomic survey (Fig. S8). The reductive acetyl-CoA pathway (Wood–Ljungdahl) and the methane metabolism (Fig. S9) were present but incomplete. The most prominent pathway observed was the 3-hydroxypropionate bi-cycle. In each turn of this cycle, three bicarbonate molecules are converted into one pyruvate. This route yields valuable intermediates for biosynthesis such as acetyl-CoA, glyoxylate and succinyl-CoA. When bicarbonate is dissolved in highly pure deuterium oxide, a probably very high ratio of deuterated bicarbonate (DCO_3_) to regular bicarbonate (HCO_3_) is expected to constitute a major cause for deuterium incorporation, mainly driven by the enzymatic activity of the widely present acetyl-CoA carboxylase [60].

Another potential carbon source for growth could come from plastic degradation, as the bacterial communities have survived for 30 years in HDPE containers. Within this context, we ubiquitously identified bacterial polyethylene-degrading genes (*alkB*1-*alkB*2 [61] and *alkM* [62]; Table S5). We also detected mono- and poly-ethylene terephthalate hydrolases (Fig. S10). A correlation was observed between the import/oxidation of polyamines (indicators of decaying living matter [63]) and the terephthalate-degrading functions (Fig. S10). This suggests a coordinated utilization of biomass- and plastic-derived carbon under nutrient limitation. A detailed analysis is currently underway to investigate bacterial activity on the polymer surface inside the plastic drums and to detect signs of metabolic activity or polyethylene degradation in the heavy water.

#### Nitrogen metabolism

Nitrogen sources are often scarce in oligotrophic environments, so we searched for nitrogen-fixing functions. We identified ten distinct ORFs annotated as *nifH* (dinitrogenase reductase), though none of the ORFs corresponded to *nifD* or *nifK* genes, which pertain to molybdenum-iron nitrogenases. These *nifH* genes belonged to three unknown species within known genera (Fig. S2a). However, upon deeper analysis of these ORFs, it was apparent that they corresponded to the *nifH* Cluster IV, which is widely recognized as non-functional for nitrogen fixation (Fig. S2b [64]). The sequences frequently clustered with the outgroup with high bootstrap values, making it unlikely that these were involved in nitrogen fixation. Thus, we cannot definitively identify a primary source of nitrogen. However, despite the uncertain origin of the nitrogen compounds in our samples, the nitrogen cycle was extensively represented. Genes involved in key nitrogen redox processes were clearly present (Fig. S11), including nitrate transporters (*nrt*). Thus, denitrification, assimilatory nitrate reduction (for protein synthesis) and dissimilatory nitrate reduction (for energy production and ammonia formation) pathways were enabled. Aerobic and anaerobic ammonia oxidation processes (anammox) were mostly irrelevant, except for the final step of nitrification. Genes involved in nitroalkane metabolism genes were also detected. Finally, urea metabolism was ubiquitous, with the *ureC* (marker for urease) and *urtB* (marker for urea transporter) genes abundant across samples (Fig. S12).

#### Sulphur metabolism

Prokaryotic sulphur metabolism was also widely represented in the analysed metagenomes (Fig. S13). The Sox system, responsible for sulphur oxidation, was both widespread and abundant, with sulphate thiolation being found in all samples. Extracellular transporters for sulphate, taurine and alkenesulfonate were also found in high abundance. Genes encoding enzymes that mediate the reduction of sulphur compounds to sulphite and to sulphur were prevalent. In addition, the complete set of enzymes required for the assimilatory sulphate reduction (sulphate to sulphide) was present. Based on the genes found, acetate may serve as an intermediary for carbon fixation, while l-serine could be used for l-cysteine and l-homocysteine conversion. l-Homoserine may be further catabolized to succinate. Other metabolic intermediates, such as acetaldehyde, could be generated from methylthiopropionate catabolism.

### Diversification of sequences was widely maintained in pure heavy water

Finally, to interrogate whether sequence richness (i.e. the number of sequence variants per gene name) was constrained by heavy water, we fitted an empirical model to measure the contribution of the gene’s abundance per taxon to the total number of sequence variants (see Methods). Since the model follows a power law, we estimated how quickly variant-richness grows with ORF abundance by examining the γ parameter (slope). We summed unique gene variants for each taxon and environment, plotting these sums on the Y-axis of a log-log regression (Fig. 1a).

**Fig. 5.**
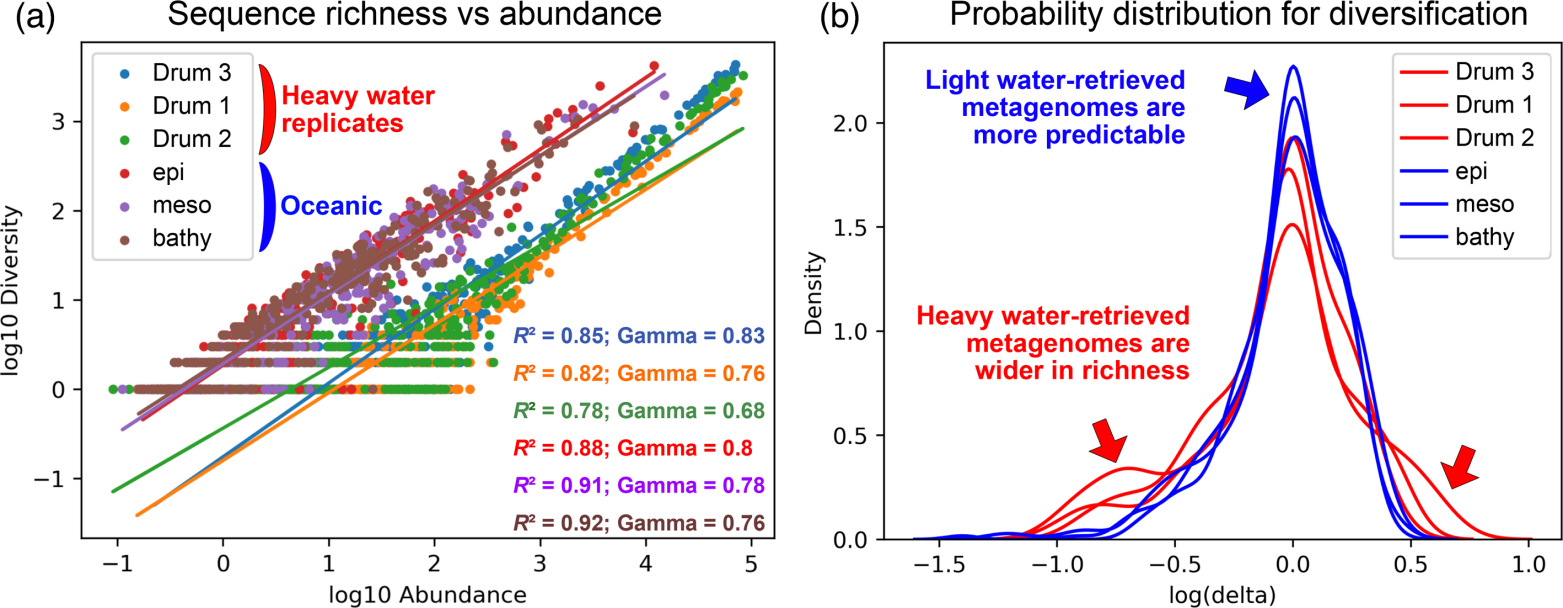
Representation of the number of sequence variants per taxon in pure heavy water vs oceanic environments. (**a)** Calculation of the expected sequence richness per taxon. The X-axis represents total abundances. Each colour in the plot corresponds to an environment, with points for each taxon (species) associated with any gene, capturing the growth of unique variants with abundance. All unique ORFs (assigned to a taxon and to a sample) are considered, together with the sum of their corresponding abundances. The unique sequence richness log for each combination is compared with the logarithm of their summed abundances. With the residuals from the log-log regression, we estimate the expected richness for a given abundance level in each context. Thus, the value of gamma (slope) reflects how much the number of distinct sequences grows in favour of their abundance. We observed that the heavy water purity level was not limiting the observed diversity of sequences. Number of points (taxa) per environment: container 3, *n*=394; container 1, *n*=319; container 2, *n*=359; epipelagic, *n*=308; mesopelagic, *n*=358; bathypelagic, *n*=351. (**b)** Probability distribution of the unexpected sequence richness. Non-zero values on the X-axis indicate deviations from expected sequence richness. Higher values on the Y-axis correspond to a greater probability of encountering a taxon with the given X value.

For comparison with a representative oligotrophic water system, we analysed oceanic metagenomes from the Malaspina dataset [31]. It is observed that the intercept for oceanic metagenomes was much higher, mainly because we use a subset of nitrogen-related genes with their characteristic abundance ratio, but whose richness is equally deducible from abundance. Yet, the slopes were not significantly different (γ_D₂O_ = 0.757 ± 0.072; γ_ocean_ = 0.78 ± 0.023), indicating that high deuterium concentrations at least did not constrain sequence diversification, hinting at the feasibility of gradual genotypic adaptation to heavy water. Moreover, when calculating δ values (δ=1 indicates that the richness of sequence variants *per taxon–environment* is deduced from the sum of their abundance; Methods), the distribution of unexpected richness values can be inferred (Fig. 1b). This probability distribution was found to be wider when compared to the oceanic metagenomes, indicating a higher likelihood of finding taxa with lower-than-expected variant richness (suggesting preservation of sequence variants) and taxa with higher-than-expected variant richness (suggesting positive selection) in the heavy water-retrieved metagenomes.

## Conclusions

This work is pioneering in providing a comprehensive description of metagenomes sequenced from unprecedented artificial environments with extremely high deuterium concentrations and in demonstrating the growth of long-term heavy water-retrieved bacteria. Its primary objective was to explore the potential for bacterial adaptation to highly pure heavy water. Our findings suggest that bacteria, and by extension biological systems, are capable of genotypic adaptation to a fully deuterated environment. Several lines of compelling evidence support this conclusion: (i) viable bacteria were found after long-term storage in pure heavy water; (ii) feasible metabolisms were inferred from the predicted genes despite the extreme deuterium concentrations; (iii) diversity of bacterial lineages was not strongly constrained by high deuterium concentrations, although the average gene length and genome sizes were reduced compared to their closest known relatives; (iv) transposases and DNA repair genes were overrepresented; (v) the most abundant proteins, particularly IS3-like transposases, underwent substantial changes in sequence and aa composition, likely to reduce energy requirements; (vi) the estimated $k_{a}/k_{s}$ values for IS3-like transposases indicated positive selection; and (vii) rather than being hindered, protein diversification was found to be more extensive than in other water oligotrophic systems, such as oceanic microbiomes.

## Supplementary material

10.1099/mgen.0.001414 Uncited Supplementary Material 1.

## Abbreviations

AA: amino acid
CDSs: coding sequences
TPM: transcripts per million
FPM: features per million
HDPE: high-density polyethylene
MAGs: metagenome-assembled genomes
