# Supplementary material for: Metagenomic analysis of heavy water-adapted bacterial communities
Source: Microb Genom. 2025 May 29;11(5):001414. doi: 10.1099/mgen.0.001414 (PMC12171019; doi:10.1099/mgen.0.001414)
Supplement: Uncited Supplementary Material 1. [file mgen-11-01414-s001.pdf]

Metagenomic analysis of heavy water-adapted bacterial communities

Rivas-Santisteban *et al.*, 2025

Supplementary Information

Mechanistic basis for the "deuterium-induced differential mutagenesis" hypothesis

Genotypic adaptation would be favored by a strong mutagenic effect of deuterium oxide (increase of the mutational load, μ), leading to fixation of new isoforms with greater fitness (Mosin, 1999). However, heavy water was not mutagenic on recessive and dominant lethal marks in multicellular eukaryotes (Bray, 1962). Regarding prokaryotes, Flaumenhaft and Katz grew E. coli in 99.6% deuterium oxide with fully deuterated substrates, finding a reduced mutation rate after UV irradiation compared to non-deuterated organisms (Flaumenhaft, 1967). The latter may be explained by the radioprotective effect of deuterium (Michell, 1987; Laeng, 1991). Yet, previous observations from De Giovanni (De Giovanni, 1961) add complexity to the issue: some bacterial loci showed rather variable mutation rates (regardless of whether the base was deuterated or not) when exposed to deuterium, and mutagenicity was more acute when its concentration was higher (>50\%). The induced/spontaneous mutation ratios were loci and strain specific, the lowest being 0.25x (i.e. deuterium *prevented* mutations), and the highest, 52.2x (i.e. deuterium *caused* mutations). In addition, strong morphological distortions were observed after merely eight divisions – were described as "monster cells" due to cell-size increase (2x to 10x, with a 25% of viability) in response to deuterium treatments. Finally, no strain was able to fully adapt to isotopic effects, as growth was found to be systematically greater when re-exposed to standard protium conditions. However, this observation was made in immediate generations, matching the idea that some genes were mutating more because of the molecular mechanisms described in the main text (eg. peroxide accumulation due to inhibition of the *kat* genes), while others were in fact over-maintained (as nucleotide excision and repair are upregulated in presence of deuterium).

Supplementary files

File S1: FASTA file of the colony-recovered 16S sequences.

File S2: FASTA file of the IS3-like sequences used for the analyses concerning transposases.

Supplementary tables

Table S1: Contig summaries (SqueezeMeta's output).

Table S2: Taxonomic proportion of ORFs found in each container.

Table S3: SqueezeMeta's co-assembly summaries.

Table S4: Proportion of aa in both deuterium-retrieved and common IS3-like transposases. First set of columns consist of absolute counts, while second tier of columns are relative values.

Table S5: Subset of the ORFs related to plastic degradation.

Element	Concentration (µg/L)
Si	9837.55
Li	3231.66
K	2610.56
Na	454.23
B	300.84
Ca	185.45
Al	23.05
Br	22.71
Mg	11.35
Cr	8.70
Pb	5.30
Cu	4.47
Kr	4.06
Se	2.80
Ti	1.42
Pt	1.33
Sr	0.69
Ba	0.62
Fe	0.37
Mo	0.33
As	0.32
Ni	0.25
Rb	0.22
W	0.17
Mn	0.08
Sc	0.08
Cd	0.07
Ag	0.06
Bi	0.06
Ga	0.05
Cs	0.04
Au	0.04
In	0.01
Tl	0.01
Zr	0.01

Figure S1: Main impurities found in the heavy water inside the containers. Elements not detected by ICP-MS (inductively coupled plasma mass spectrometry) are not shown.

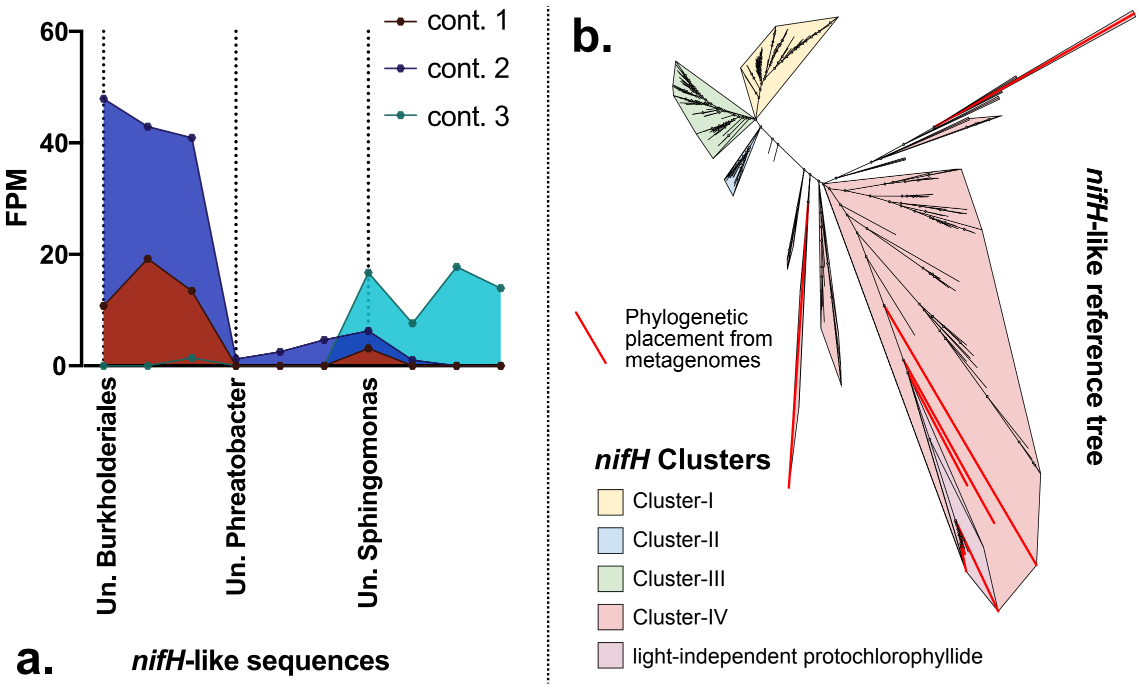

Figure S2: Nitrogen fixation cannot be determined in the metagenomes with the available evidence. A. ORFs related to nifH and their abundances (FPM) were exclusively found over three unclassified taxa. Abundances were higher in the most deuterated system. B. Placement of nifH-like ORFs into a nifH reference tree. Red bars represent phylogenetic distances from our metagenomic retrieved queries to the reference sequences of nifH. Cluster IV is widely regarded as non-functional. We therefore discarded nitrogen fixation as the most probable form of nitrogen acquisition.

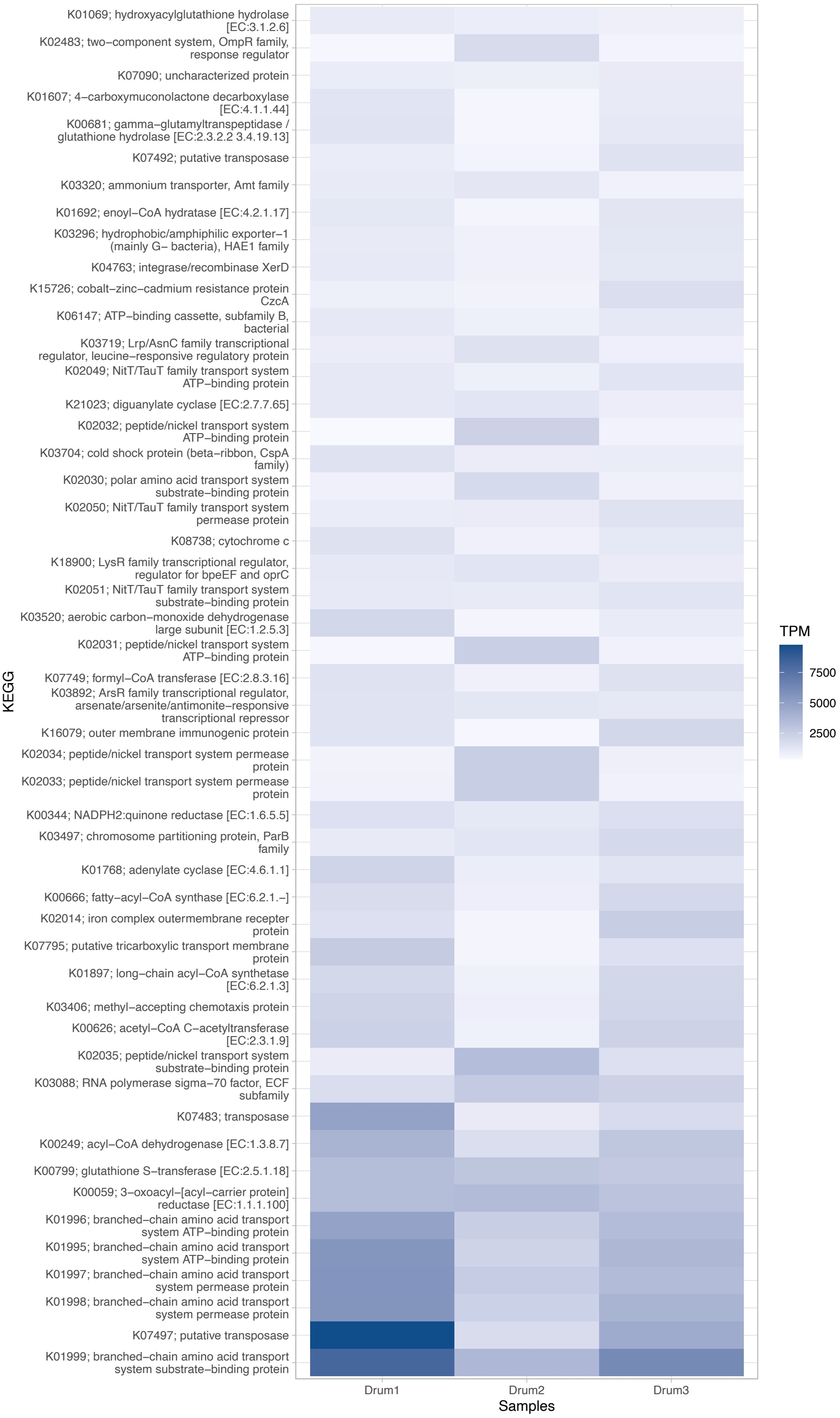

Figure S3: Top 50 functional annotations (KEGG) of metagenomes retrieved from highly pure deuterium oxide samples. TPM is equal to Transcript, or Features, Per Million.

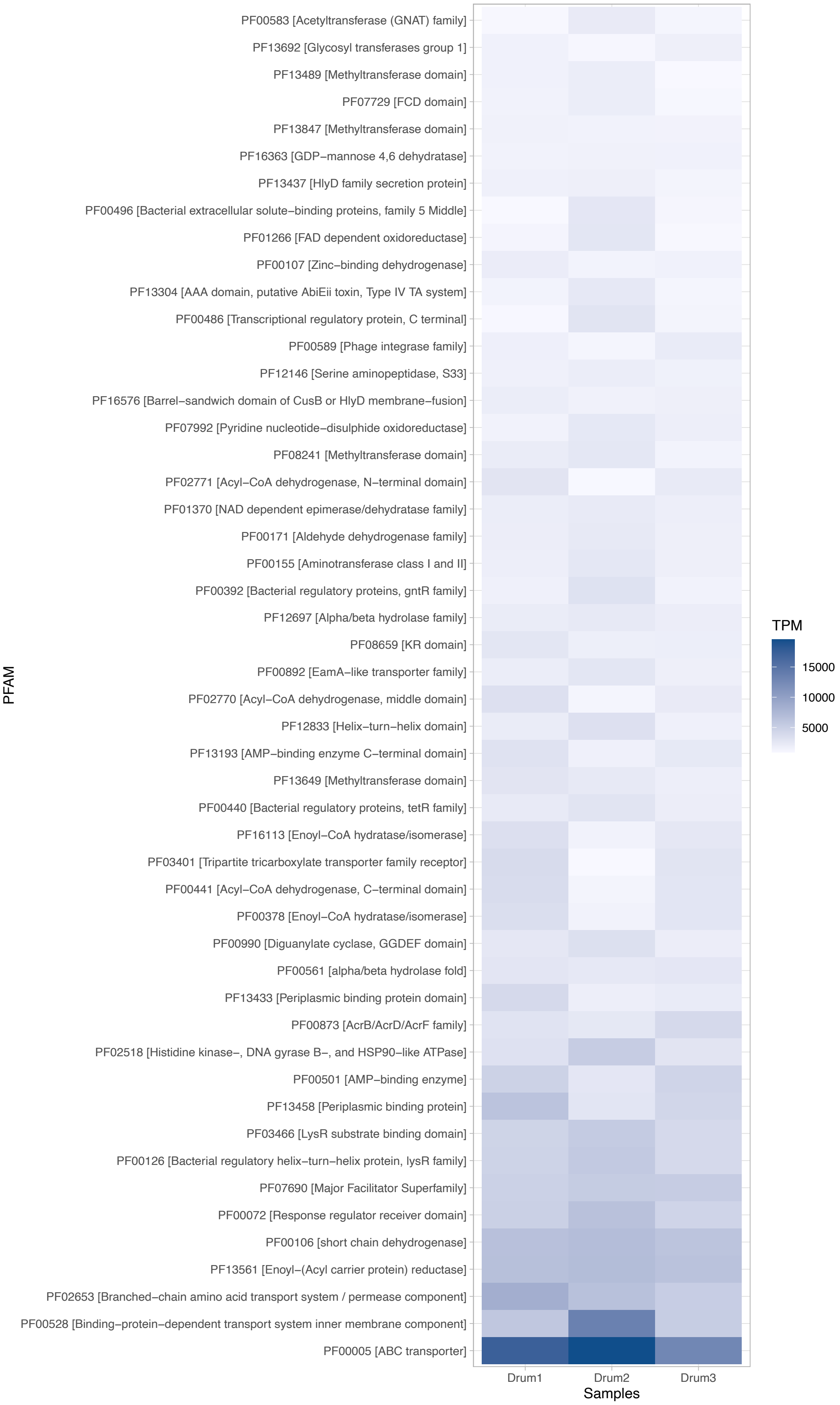

Figure S3.1: Top 50 functional annotations (PFAM) of metagenomes retrieved from highly pure deuterium oxide samples. These annotations are broader in function and were not used for the analyses described in the main text.

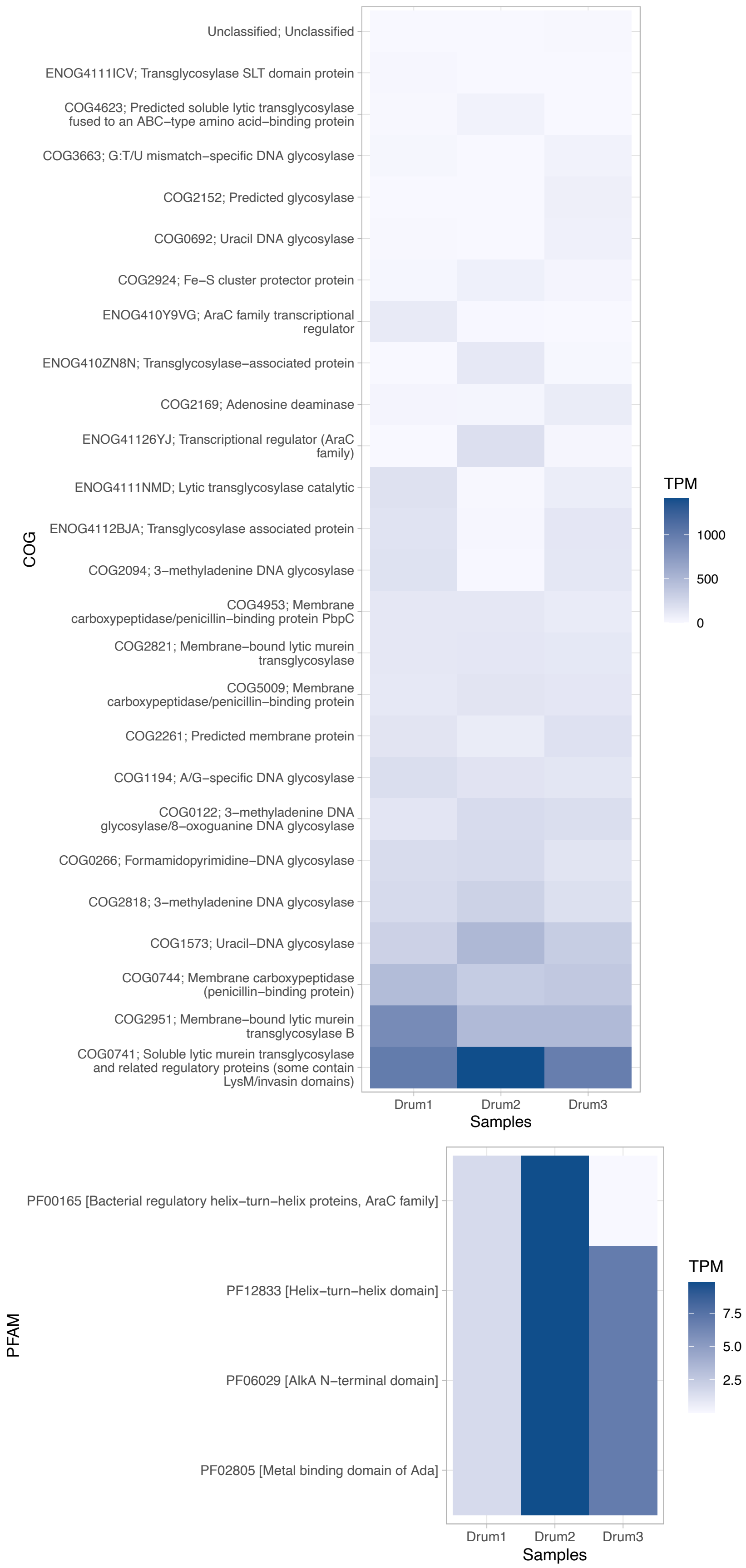

Figure S4: Top: COGs related to DNA repair found within high deuterium metagenomes were found in the three containers. Bottom: *ada-alkA*/*araC* related annotations found were consistent across containers.

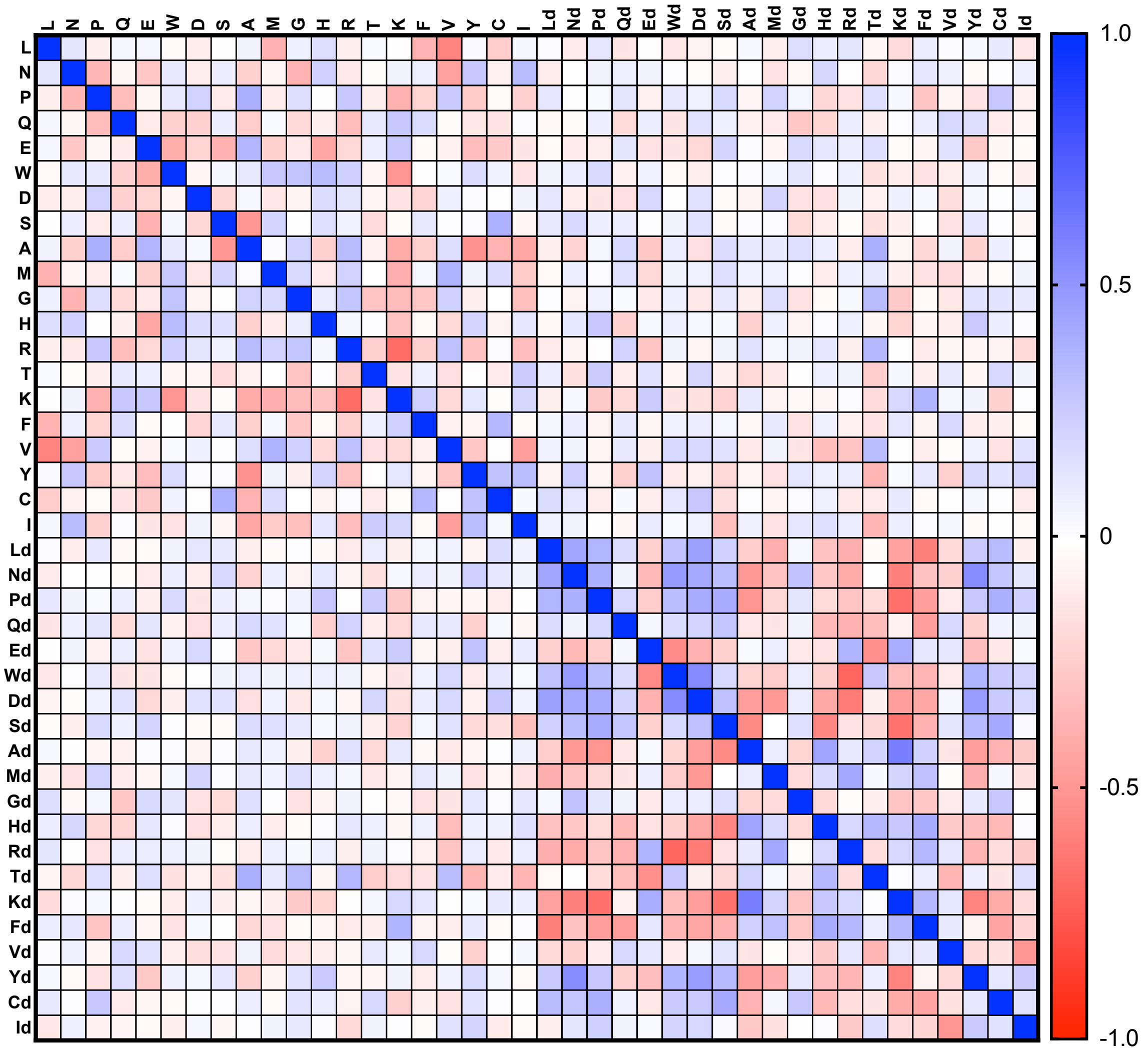

Figure S5: Correlation matrix for amino acid usage of common-sources IS3 transposases (upper left, common amino acid symbols) and deuterium-retrieved IS3 transposases (right bottom, modified amino acid symbols). Color scale represent the polarity and strength of the correlation. The correlation between frequencies of the same amino acid is always 1.0 (the blue diagonal). Frequencies of each group of transposases autocorrelated with more strength (intensity and disparity of colors) compared to the amino acid frequency between groups (left bottom & upper right). This result strongly suggests that amino acid usage of the closest-known IS3 transposases is dissimilar.

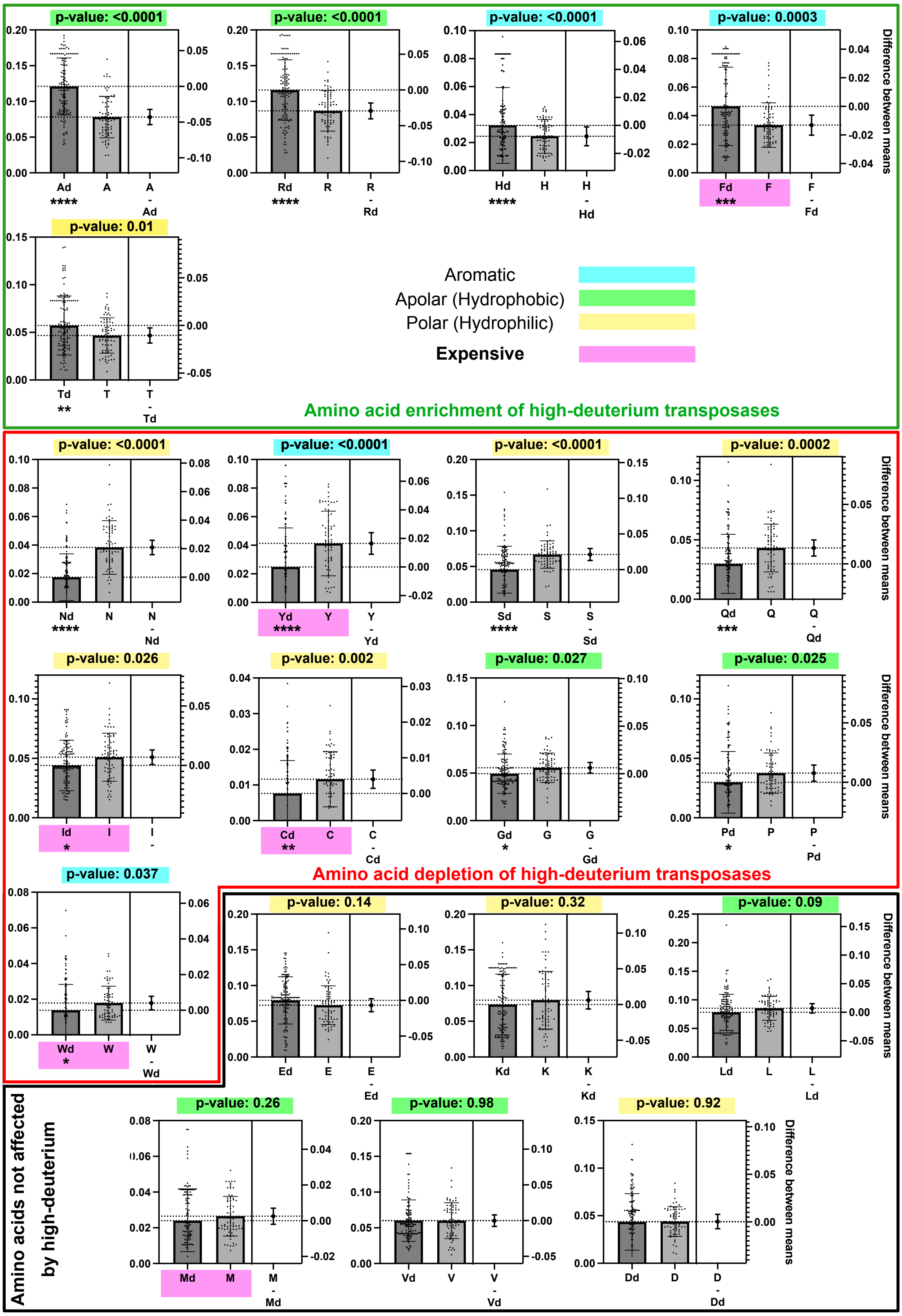

Figure S6: Unpaired t-test for amino acid frequency among IS3-like transposases. Amino acid composition changed drastically in the deuterium-retrieved transposases (n=129) compared to those sequenced within natural sources (n=70). The Y-axis in each graph represents the specific amino acid frecquency in each transposase. In particular, a remarkable enrichment of particular aromatic (blue) and hydrophobic (green) amino acids is observed, as opposed to an impoverishment of expensive (pink, Akashi et al. 2002) and polar (yellow) amino acids.

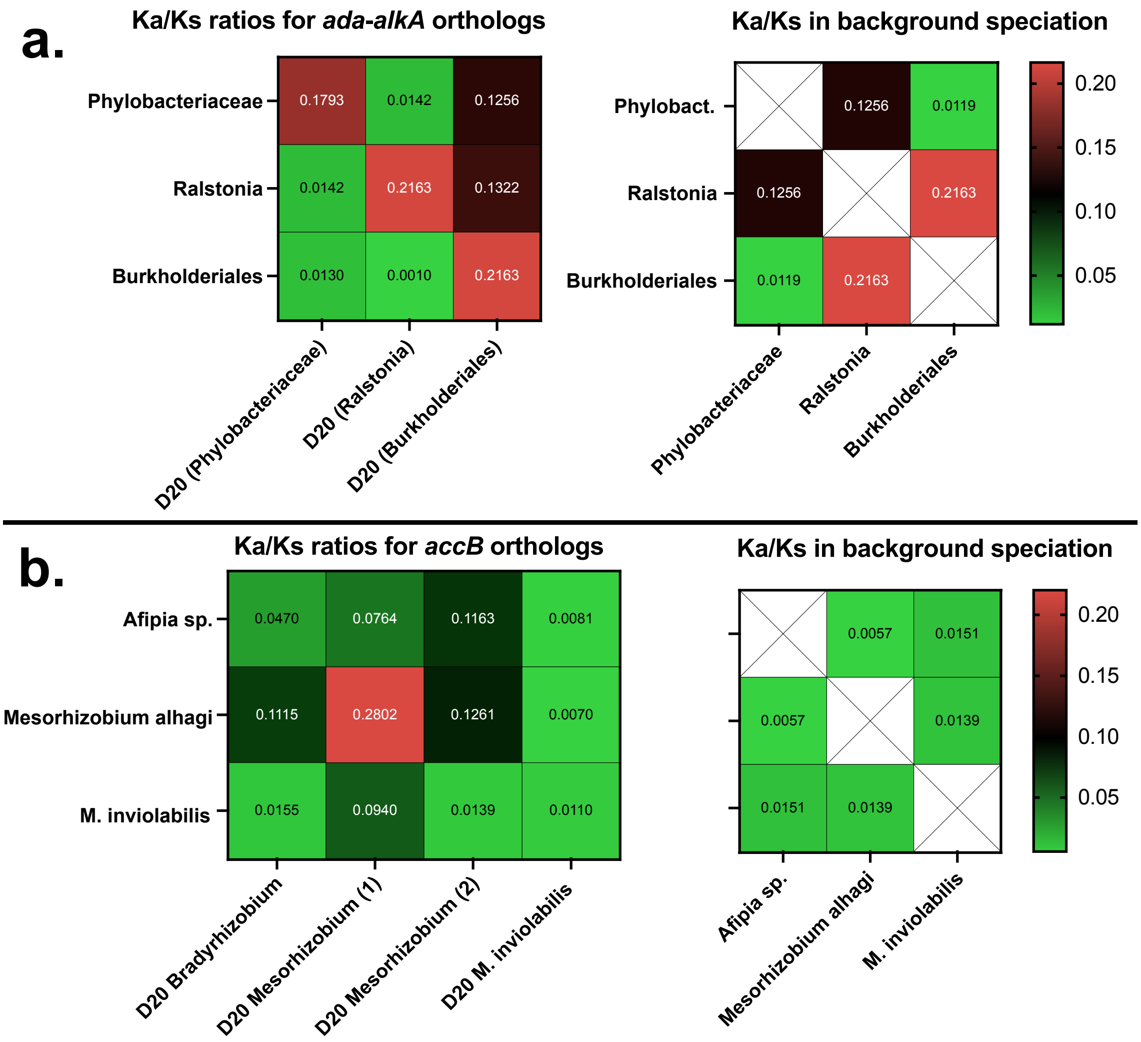

Figure S7: ka/ks ratios in other abundant genes. A. Relaxation of the purifying selection was not different from the expected from speciation in *ada*/*alkA* most abundant ORFs. B. Deuterium-retrieved *accB* orthologs (carboxylases incorporating hydrogen carbonate) were constrained too. However, the loose purifying selection in some of the proteins was not explained by the speciation processes (right). Note how the color scale differed from the one presented in the main text (Fig. 3) to improve the visualization of the adaptative trends between these conserved orthologs.

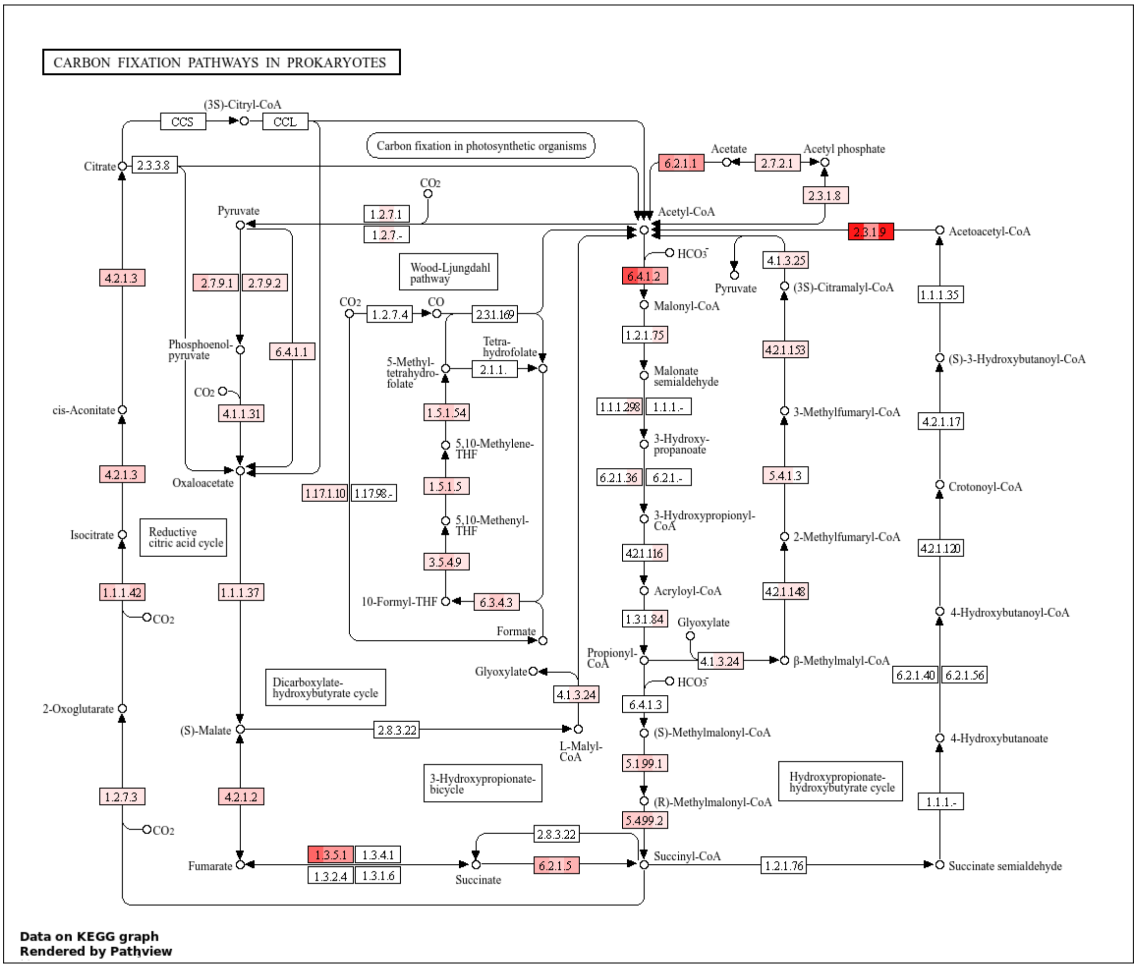

Figure S8: Representative diagram of the main microbial functions involving carbon fixation pathways in heavy water-retrieved metagenomes (KEGG pathways, Zhang, J. D., & Wiemann, S., 2009). Red is a relative indicator of abundance. As shown, several carbon fixation pathways in prokaryotes were present in high-deuterium environments. The 3-Hydroxypropionate bi-cycle was the most abundant, making the incorporation of DCO3- into malonyl-CoA and derivates a feasible mechanism of deuteration of biosynthetic molecules. Each colored partition represents a sample.

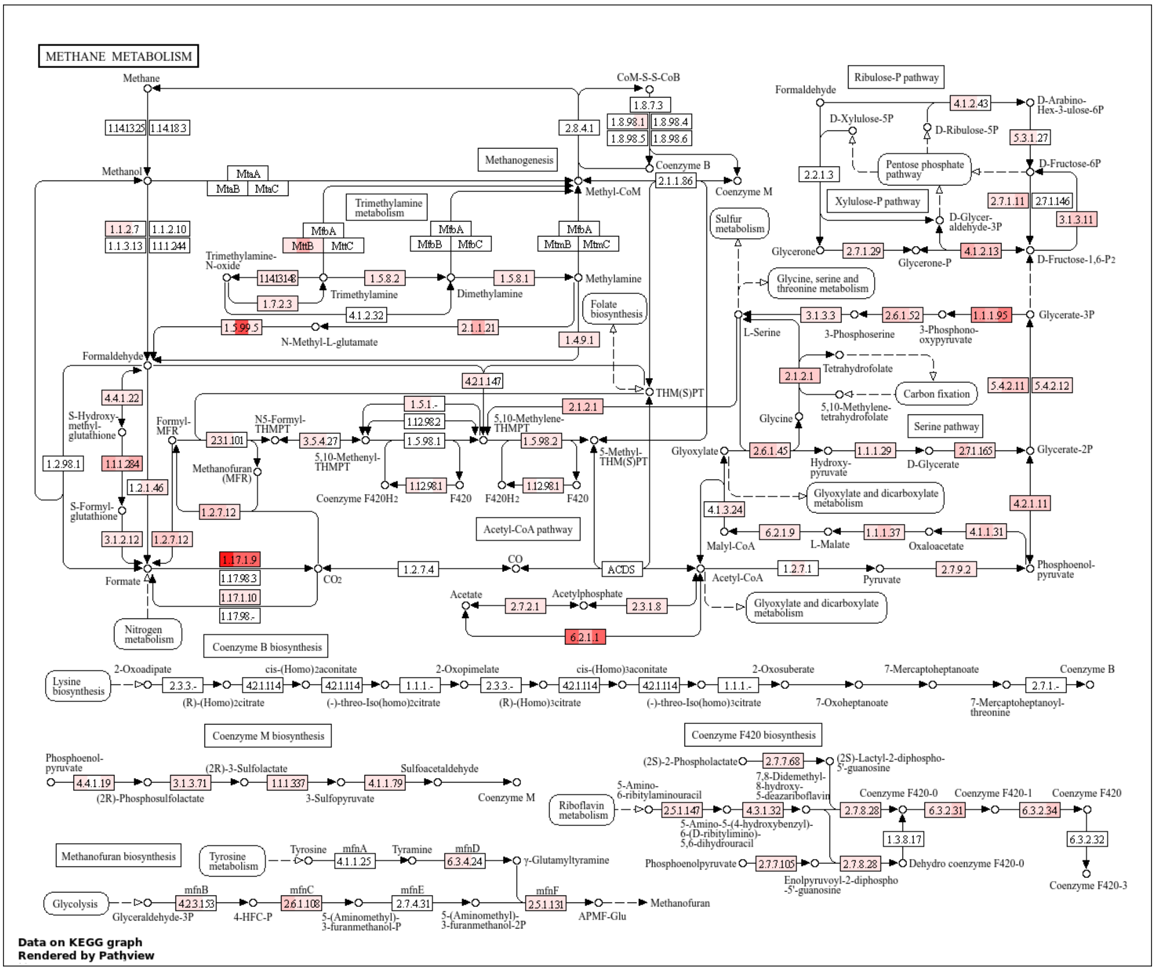

Figure S9: Methane metabolism in heavy water-retrieved metagenomes (KEGG pathways, Zhang, J. D., & Wiemann, S., 2009). It was only partially identified within our samples. However, the identified genes were consistent, as they were mostly common to all three replicates. Each colored partition represents a sample.

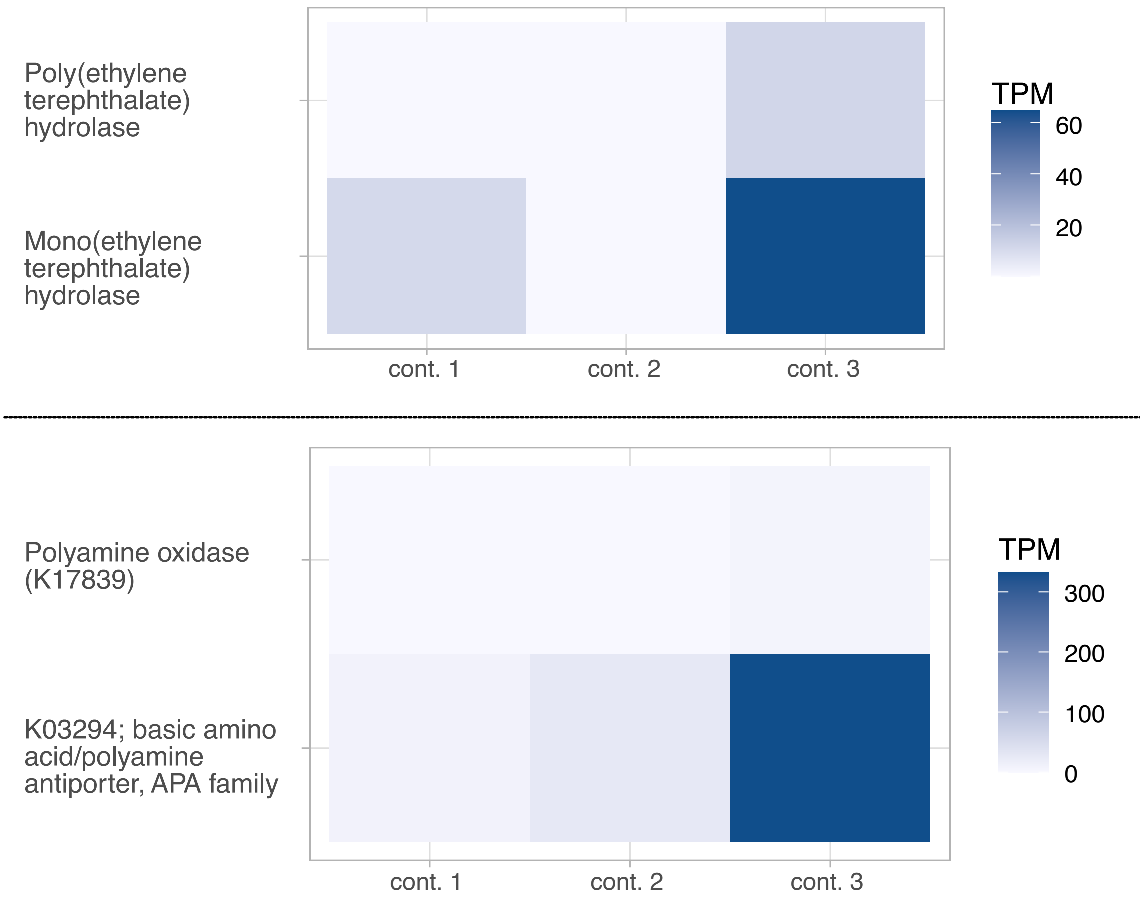

Figure S10. Top: Relative abundance of the PET-exploiting functions found. Bottom: Polyamine-exploiting functions found. Poly- and mono-ethylene terephtalate hydrolases strongly correlated to polyamine oxidation/uptake annotation presence.

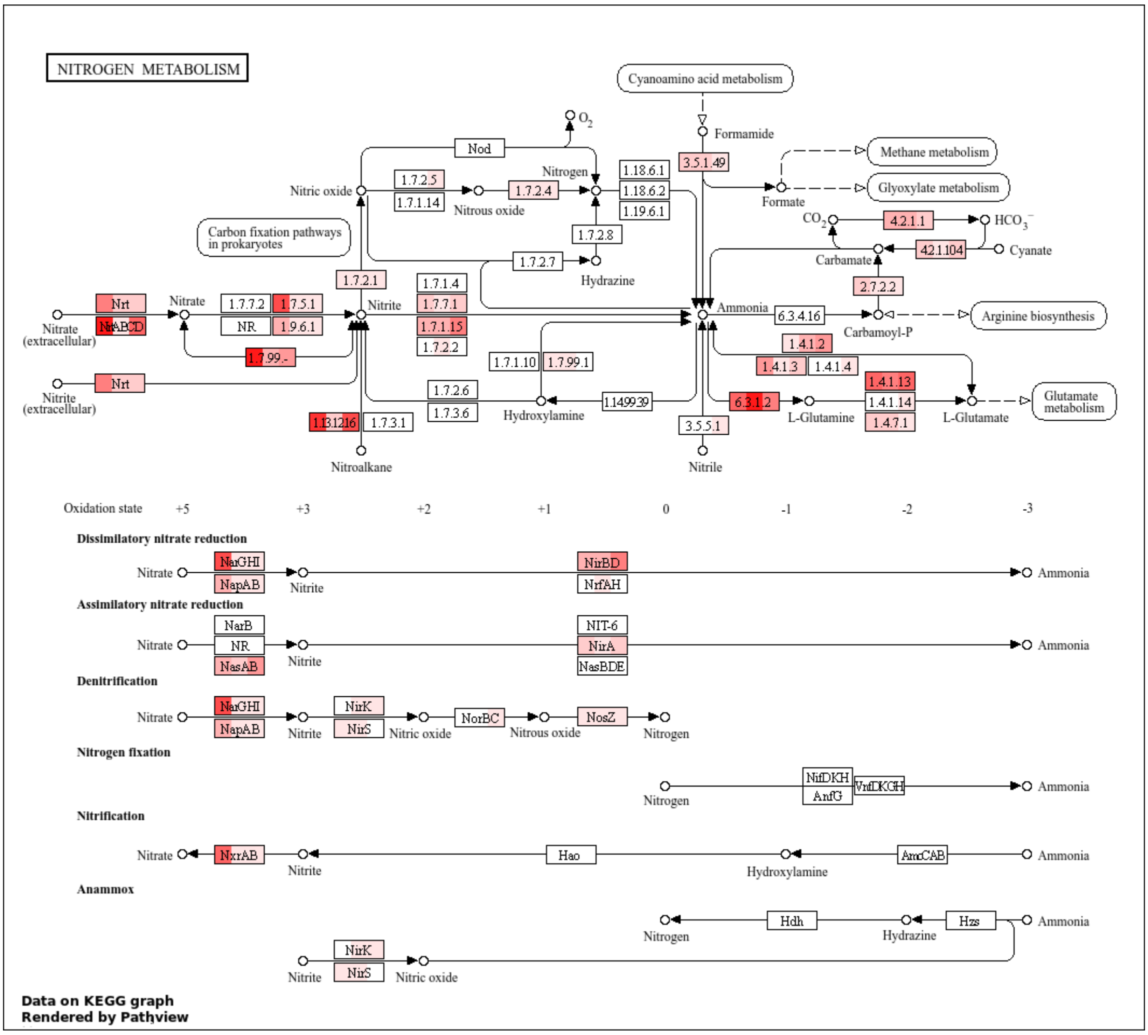

Figure S11: Representative diagram of the main microbial functions involving nitrogen showed that nitrate and amino acids are used for assimilatory/dissimilatory pathways (KEGG pathways, Zhang, J. D., & Wiemann, S., 2009) in heavy water-retrieved metagenomes. Ammonia would be produced through both routes. There is also evidence for denitrification and the last step of nitrification. Each colored partition represents a sample.

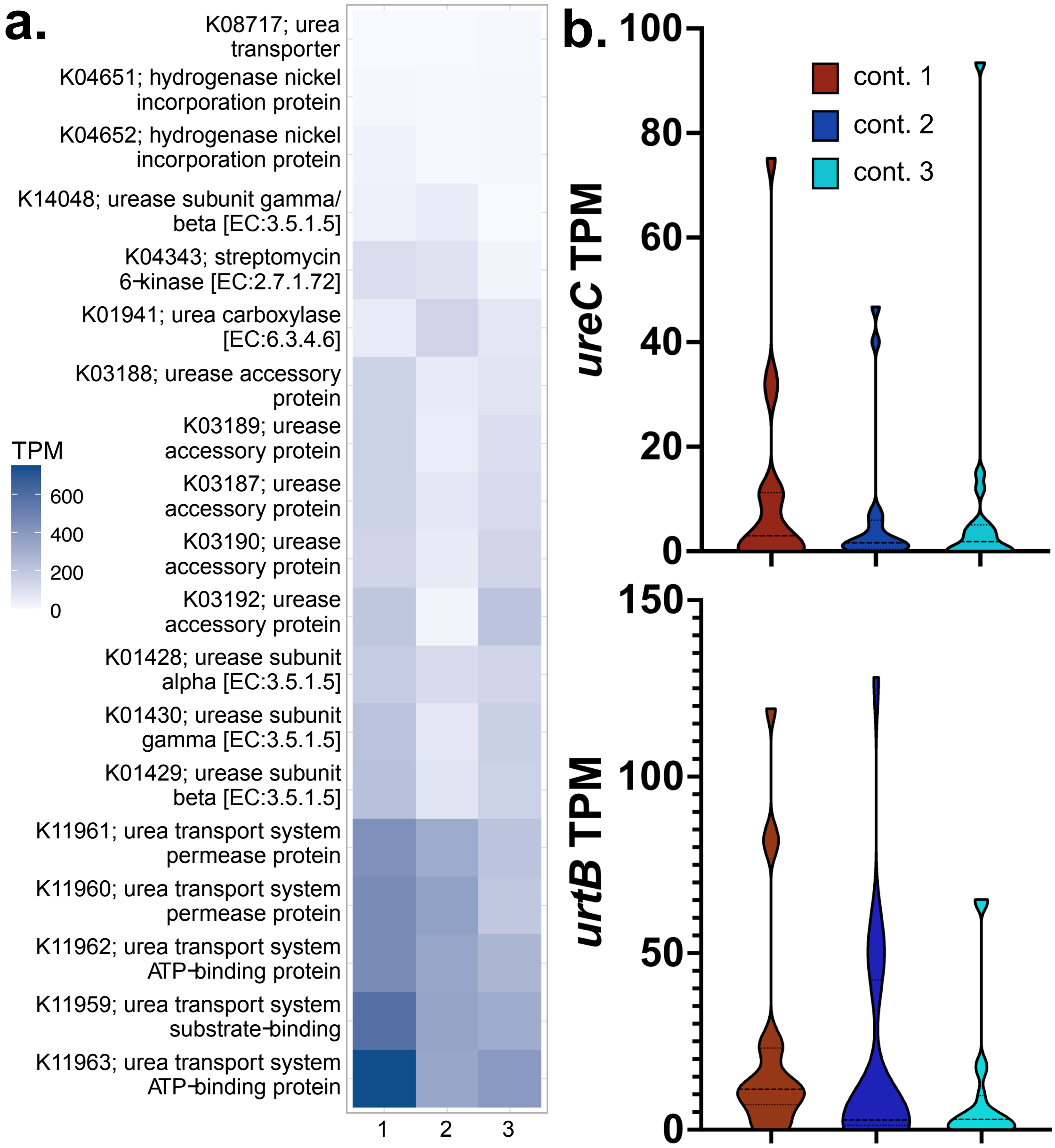

Figure S12: Urea-related functions in heavy water-retrieved metagenomes. These were systematically abundant in all deuterated containers. A. Summed abundances of urea-related metabolism were higher in the most deuterated system, although total ORFs count was significantly lower for this sample. B. Abundances (TPM) for *ureC* (urease) and *urtB* (urea transporter) genes. Urea carboxylase (*uca*) was not found.

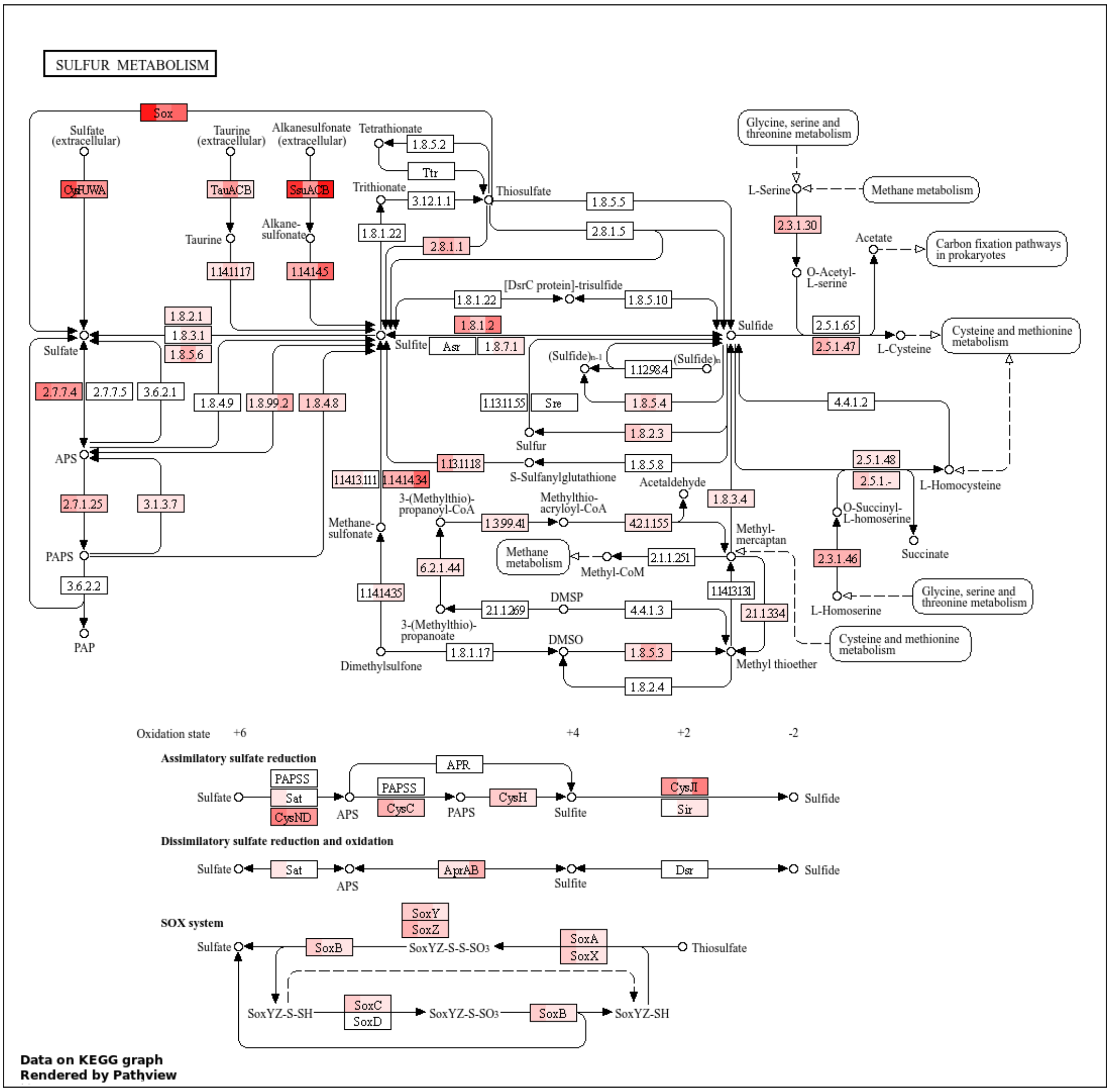

Figure S13: Main prokaryotic sulfur pathways (KEGG pathways, Zhang, J. D., & Wiemann, S., 2009) in heavy water-retrieved metagenomes. Sulfur metabolism was globally identified within our samples and appeared to recapitulate the distinctive taxonomic composition of each community.
